# Longitudinal trimodal imaging of midbrain-associated network degeneration in Parkinson’s disease

**DOI:** 10.1038/s41531-022-00341-8

**Published:** 2022-06-22

**Authors:** Kenan Steidel, Marina C. Ruppert, Andrea Greuel, Masoud Tahmasian, Franziska Maier, Jochen Hammes, Thilo van Eimeren, Lars Timmermann, Marc Tittgemeyer, Alexander Drzezga, David J. Pedrosa, Carsten Eggers

**Affiliations:** 1 Department of Neurology, University Hospital of Marburg, Marburg, Germany; 2 Center for Mind, Brain and Behavior—CMBB, Universities Marburg and Gießen, Marburg, Germany; 3 Institute of Systems Neuroscience, Medical Faculty, Heinrich Heine University Düsseldorf, Düsseldorf, Germany; 4 Institute of Neuroscience and Medicine, Brain & Behaviour (INM-7), Research Centre Jülich, Jülich, Germany; 5 Department of Psychiatry, University Hospital Cologne, Medical Faculty, Cologne, Germany; 6 Multimodal Neuroimaging Group, Department of Nuclear Medicine, Medical Faculty University Hospital Cologne, Cologne, Germany; 7 Department of Neurology, Medical Faculty and University Hospital Cologne, University Hospital Cologne, Cologne, Germany; 8 German Center for Neurodegenerative Diseases (DZNE), Bonn-Cologne, Germany; 9 Max Planck Institute for Metabolism Research, Cologne, Germany; 10 Cluster of Excellence in Cellular Stress and Aging Associated Disease (CECAD), Cologne, Germany; 11 Cognitive Neuroscience, Institute of Neuroscience and Medicine (INM-2), Research Center Jülich, Jülich, Germany; 12 Department of Neurology, Knappschaftskrankenhaus Bottrop, Bottrop, Germany

**Keywords:** Parkinson's disease, Neuroscience

## Abstract

The prevailing network perspective of Parkinson’s disease (PD) emerges not least from the ascending neuropathology traceable in histological studies. However, whether longitudinal in vivo correlates of network degeneration in PD can be observed remains unresolved. Here, we applied a trimodal imaging protocol combining 18F-fluorodeoxyglucose (FDG)- and 18F-fluoro-L-Dopa- (FDOPA)-PET with resting-state functional MRI to assess longitudinal changes in midbrain metabolism, striatal dopamine depletion and striatocortical dysconnectivity in 17 well-characterized PD patients. Whole-brain (un)paired-*t*-tests with focus on midbrain or striatum were performed between visits and in relation to 14 healthy controls (HC) in PET modalities. Resulting clusters of FDOPA-PET comparisons provided volumes for seed-based functional connectivity (FC) analyses between visits and in relation to HC. FDG metabolism in the left midbrain decreased compared to baseline along with caudatal FDOPA-uptake. This caudate cluster exhibited a longitudinal FC decrease to sensorimotor and frontal areas. Compared to healthy subjects, dopamine-depleted putamina indicated stronger decline in striatocortical FC at follow-up with respect to baseline. Increasing nigrostriatal deficits and striatocortical decoupling were associated with deterioration in motor scores between visits in repeated-measures correlations. In summary, our results demonstrate the feasibility of in-vivo tracking of progressive network degeneration using a multimodal imaging approach. Specifically, our data suggest advancing striatal and widespread striatocortical dysfunction via an anterior-posterior gradient originating from a hypometabolic midbrain cluster within a well-characterized and only mild to moderately affected PD cohort during a relatively short period.

## Introduction

Parkinson’s disease (PD) is a neurodegenerative disorder predominantly affecting people from middle to older ages. Although specific degeneration of dopaminergic neurons in the substantia nigra (SN) is the hallmark of the disease, processes beyond the basal ganglia fundamentally shape its heterogeneous phenotype. In their seminal work Braak et al.^1^ proposed ascending Lewy body neuropathology, which gave rise to the currently prevailing network perspective on PD. Gradients of misfolded proteins along the diseased brain’s neuronal connectome are traceable^2–4^, which support the idea of prion-like propagation^5^ similar to the one after *α*-synuclein inoculation in murine parkinsonian models^6^.

A more widespread circuit dysfunction of surviving neural populations could be responsible for the majority of symptoms^7^, in place of isolated spatial or temporal pathology that fails to account for the multifaceted degeneration patterns from cellular to network levels. Recently, studies began to trace the presumed order in which subsystems are affected in vivo by using different imaging techniques including dopaminergic PET and neuromelanin-sensitive MRI and were able to distinguish a body-first from a brain-first subtype of PD, underscoring the great potential of multimodal imaging protocols for clarifying the involvement of specific neural pathways in disease processes^8^.

Multimodal imaging has emerged as a powerful way for investigating complex interplays in systemic neurodegenerative diseases. Combined approaches using high-resolution PET and MRI modalities have been utilized to ascertain the spatial relationships between hypometabolism and functional connectivity (FC) alterations within specific networks in neurodegenerative disorders such as Alzheimer’s disease^9^. Transferring this principle to PD, we recently highlighted striatocortical degeneration relating to nigrostriatal dysfunction via ^18^F-fluoro-L-Dopa (FDOPA) PET, 18F-fluorodeoxyglucose (FDG) PET, and resting-state functional MRI (rs-fMRI)^10^. In this context, a hypometabolic midbrain cluster as an indirect correlate of nigral degeneration in PD was traceable as a presumed starting point of network-dependent degeneration. Related studies could similarly show that cortical thinning occurs at areas functionally connected to hubs such as the SN and the posterior putamen^11,12^. The spatial pattern of cortical thinning thereby puts forward the idea of the SN as disease “epicenter” or “reservoir” in accordance with our previous findings^12^.

The importance of nigrostriatal pathway alterations in both PD and its prodromal states is further underlined by results obtained using various MRI modalities^13,14^ and multi-tracer PET^15,16^. Particularly FDOPA-PET exposed hypodopaminergic striatal signaling and gave rise to the hypothesis of an anterior-posterior striatal gradient of dopaminergic dysfunction^17^. In addition, much effort has been invested in the study of striatocortical networks, which may serve as a starting point for more widespread cortical dysfunction. In this context, the sole consideration of sensorimotor connections is seen as an oversimplification^10,16,18,19^ with a meta-analysis of rs-fMRI, suggesting disturbances beyond^20^. In our previous study, we could show that striatocortical functional decoupling of dopamine deleted areas is linked to both motor and cognitive symptoms. In addition, by means of FDG-PET, the involvement of widespread cortical regions in PD has been confirmed in longitudinal studies^21–24^.

All these observations led to the hypothesis that the spatial distribution of affected striatal regions in the course of the disease determines the functional networks affected, and that resulting disturbances could be predicted based on individually affected regions. Yet, this hypothesis awaits confirmation in longitudinal analyses. Specifically, to the best of our knowledge, the relationship between nigrostriatal dopaminergic deficits, striatocortical network dysfunction, and cortical FDG-metabolism has never been addressed longitudinally with respect to symptoms of PD patients.

To ascertain the underlying mechanisms of clinical deterioration, this study employed a multimodal imaging approach in a cohort of PD patients who were re-examined after more than one year. With special emphasis on nigrostriatal connections, we first sought to examine the longitudinal changes in midbrain metabolism and striatal dopaminergic activity. We assumed, in good accordance with existing literature, a spread of dopaminergic dysfunction to anterior striatal regions over time. In addition, we examined the relationship between longitudinal striatal dopamine deficits and striatocortical connectivity and their association with increasing disease severity over time. Our research aimed at testing the overarching hypothesis that multimodal imaging may help to establish a specific footprint of the course of PD.

## Results

### Demographic, clinical, and behavioral data

In total, 17 patients aged 67.12 ± 8.19 years were available for follow-up examinations. Patients did not differ from healthy controls in any demographic variable at baseline or at follow-up visit (Table 1). The mean disease duration at baseline was 3.8 ± 2.9 years and the intervals between visits were 13.8 ± 4.1 months. Significantly higher Unified Parkinson’s disease rating scale part III (UPDRS-III) OFF scores were observed at follow-up compared to baseline (*p* < 0.001) with tremor scores (TS) (*p* = 0.024), akinetic-rigid (ARS) (*p* = 0.001), left-body (LBS) (*p* = 0.016), and right-body (RBS) (*p* = 0.043) subscores deteriorating significantly as well. As implied by ratio of hemibody UPDRS-III subscores (LBS, RBS), asymmetric expression of symptoms (laterality) was equally distributed in the baseline cohort and did not change significantly over time. There was a strong increase in the Parkinson’s disease sleeping scale (PDSS) scores (*p* < 0.001), but no increase in the non-motor symptoms scale (NMS, see Table 1). This difference in PDSS was mainly attributable to a change in item 11 (*p* = 0.009) referring to OFF-dystonia. Notably, there was linear relation between changes in PDSS and UPDRS (*r* = 0.80, *p* < 0.001). Higher motor symptom scores were not reflected in significant differences concerning quality of life, mood, or levodopa equivalent daily dose (LEDD). Further, Mini-Mental State Examination (MMSE) and Parkinson Neuropsychometric Dementia Assessment (PANDA) ratings and the more specific neuropsychological tests (Supplementary Table 2) were comparable, indicating stable overall cognition.

**Table 1:** Demographic, clinical, and behavioral variables.

Demographics of patients and healthy controls				
	PD^a^ (mean ± SD)	HC (mean ± SD)	*p*-value	Test statistics
*n*	17	14	–	–
Age [years]	67.12 ± 8.19	64.50 ± 8.29	0.170	*t* = 1.401
Gender (f)	9	7	0.732	*X*^2^ = 0.12
Disease duration [years]	3.80 ± 2.93	–	–	–
Age at onset [years]	62.53 ± 8.87	–	–	–
Time elapsed [months]	13.82 ± 4.10	–	–	–
Clinical characteristics of patients by visit				
	Baseline (mean ± SD)	Follow-up (mean ± SD)	*p*-value	Test statistics
UPDRS-III OFF-	22.12 ± 7.17	26.53 ± 7.39	**<0.001**	*t* = −5.90
ARS	8.94 ± 4.22	12.71 ± 4.41	**0.001**	*t* = −3.98
TS	0.76 ± 1.03	1.76 ± 1.86	**0.024**	*V* = 5.00
PGS	2.00 ± 2.0	2.41 ± 2.4	0.251	*V* = 5.50
RBS	8.65 ± 3.52	9.71 ± 3.84	**0.043**	*t* = −2.20
LBS	7.94 ± 3.29	9.41 ± 3.48	**0.016**	*t* = −2.71
Laterality (right/left/equal)	8/7/2	9/7/1	0.822	*X*^2^ = 0.39
RBNTS	8.12 ± 3.16	8.76 ± 3.29	0.140	*V* = 20.00
LBNTS	7.71 ± 3.24	8.59 ± 3.04	0.120	*t* = −1.69
MMSE	28.35 ± 1.50	28.24 ± 1.89	0.859	*V* = 48.50
NMS	36.82 ± 38.20	43.06 ± 35.32	0.246	*V* = 51.50
PDSS	13.24 ± 8.88	30.53 ± 17.57	**<0.001**	*t* = −6.03
PDQ39	23.80 ± 19.43	22.61 ± 12.96	0.469	*V* = 65.00
BDI-II	8.47 ± 4.76	9.24 ± 6.30	0.650	*V* = 40.50
FOG-Q	5.12 ± 5.80	7.24 ± 6.41	**0.009**	*V* = 10.50
LEDD (mg)	375.74 ± 203.18	412.60 ± 238.39	0.280	*t* = −1.12

Demographic and clinical characteristics. Statistical analyses between Parkinson’s disease patients (PD) and healthy controls (HC) were performed using Welch’s *t*- or Mann–Whitney-*U*-tests and in the case of dichotomous variables by Chi-square test. Differences in clinical variables between baseline and follow-up were analyzed with paired *t*-tests or Wilcoxon signed-rank tests.

*p* values that are statistically significant are shown in bold.

*UPDRS-III* Unified Parkinson’s Disease Rating Scale, *PANDA* *Parkinson* Neuropsychometric Dementia Assessment, *TS* Tremor score, *NMS* Non-Motor Symptoms Scale, *PDSS* Parkinson’s Disease sleeping scale, *PDQ39* Parkinson’s Disease Questionnaire, *BDI-II* Beck’s Depression Inventory-II, *FOG-Q* Freezing of Gait Questionnaire, *LEDD* Levodopa equivalent daily dose, *RBS* right-body score, *LBS* left-body score, *ARS* akinetic-rigid score, *RBNTS* right-body non-tremor score, *LBNTS* left-body non-tremor score, *PGS* postural and gait score.

^a^Parkinson’s disease patients of fMRI cohort at baseline; HC with all modalities.

All but one patient underwent FDG-PET scanning; whereas for 15 patients FDOPA-PET was available. Demographics are summarized in Table 1.

### Progression of midbrain hypometabolism and striatal dopamine depletion

**Fig. 1:**
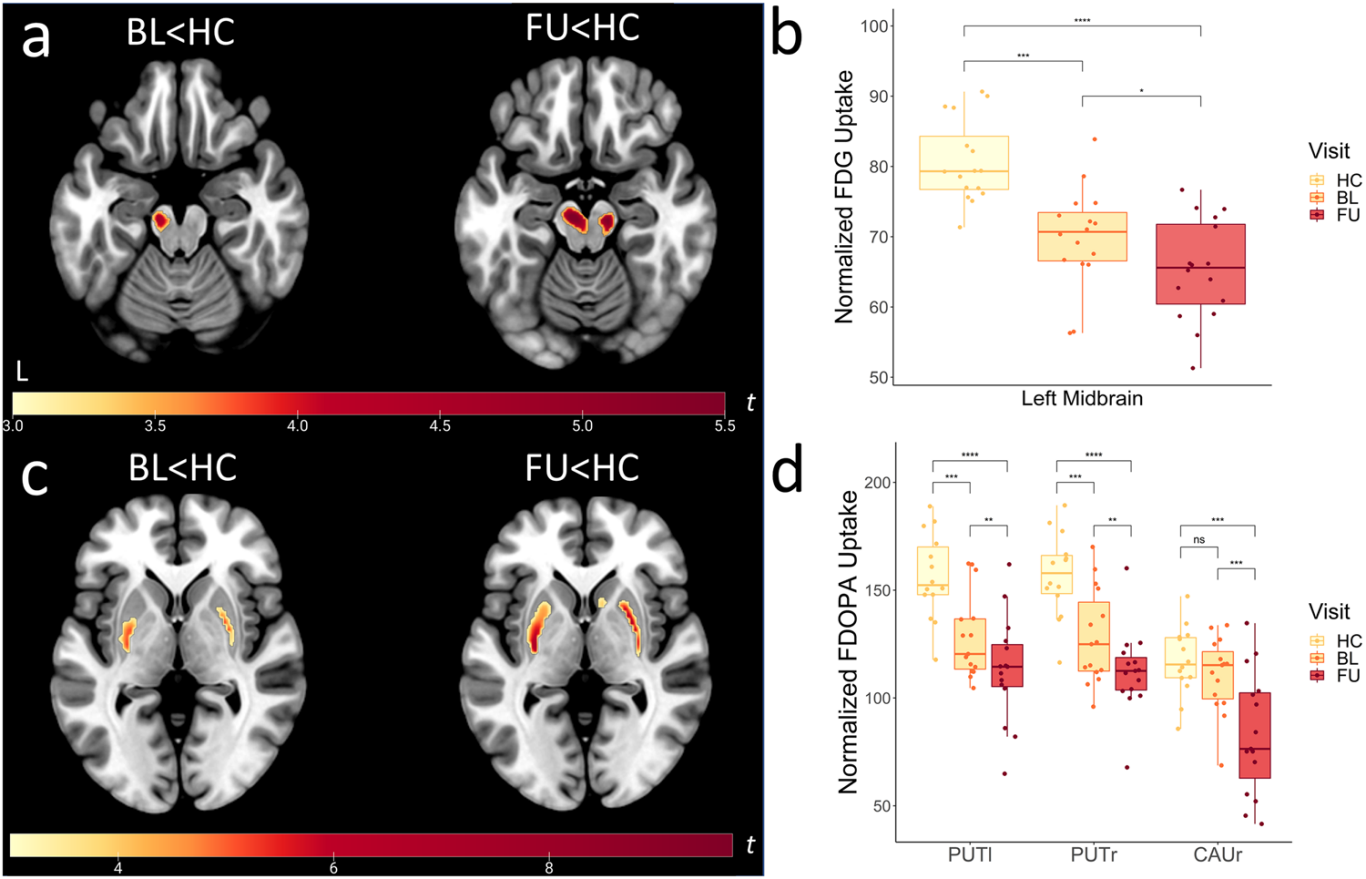
Between-group differences in FDG and FDOPA uptake versus healthy controls. T-maps were generated in SPM12 by voxel-vise *t*-tests with the above displayed contrasts. **a** Patients with Parkinson’s disease showed FDG hypometabolism at baseline and follow-up visit in comparison to healthy controls. Extracted normalized FDG uptake (**b**) and FDOPA uptake (**d**) values from respective clusters (FU < HC) are shown as boxplots with individual data points in a direct comparison between baseline, follow-up, and healthy controls (*p < 0.05, **p < 0.01, ***p < 0.001, ****p < 0.0001, central line = median, bounds of box = 25th to 75th percentile, whiskers = smallest to highest value within 1.5 times interquartile range below 25th percentile respectively above 75th percentile). **c** Patients exhibited dopaminergic deficits in the bilateral posterior putamen compared to healthy controls at both visits. Results are shown at *p*_FWE_ < 0.05 cluster-level corrected using SVC for a midbrain (**a**) or striatum (**c**) ROI. *T*-values of resulting clusters are indicated by the colorbar. Boxplots contain extracted normalized FDG and FDOPA values with proportional scaling. BL = baseline, FU = follow up, HC = healthy controls, PUTl = left putamen, PUTr = right putamen, CAUr = right caudate nucleus.

**Fig. 2:**
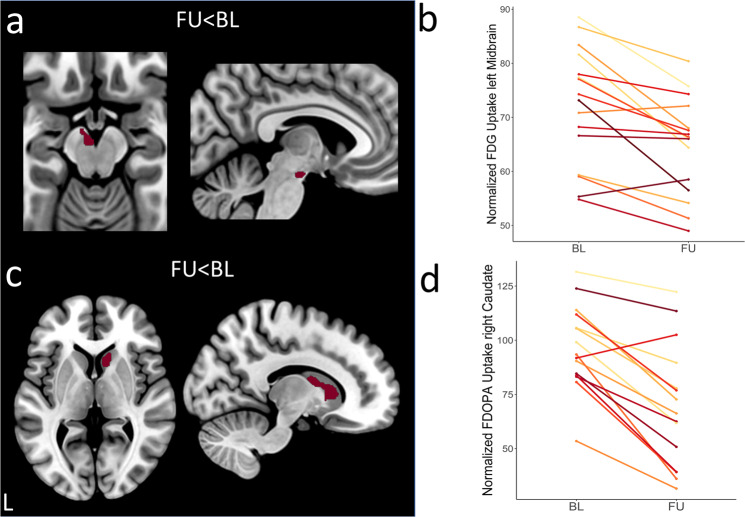
Direct comparison of FDG and FDOPA uptake between follow-up and baseline. Patients showed significantly reduced FDG uptake in the left midbrain (**a**) and significantly reduced FDOPA uptake in the right caudate (**c**) at follow-up visits compared to baseline. Both clusters are displayed in binarized form. Results were obtained by applying the above stated contrasts, thresholded at *p*_FWE _< 0.05 cluster-level corrected with SVC for midbrain (**a**) or striatum (**c**), respectively. Progression plots show the decrement of tracer uptakes between both visits for each patient individually for left midbrain FDG uptake (**b**) and right caudate FDOPA uptake (**d**). Progression plots contain extracted normalized FDG and FDOPA values with proportional scaling. BL = baseline, FU = follow up, HC = healthy controls.

Voxel-wise *t*-tests of FDG-PET scans between patients at baseline or follow-up and healthy controls with family-wise error correction (FWE) both revealed hypometabolic midbrain clusters (cf. Fig. 1a). At baseline, the cluster was constricted to the left midbrain, at follow-up it was bilateral, larger and more significant (Fig. 1a right). Exported tracer uptake values from the resulting midbrain cluster (follow-up < healthy controls) indicated gradual changes in midbrain metabolism between baseline and follow-up (Fig. 1b). Direct voxel-wise comparison between baseline and follow-up visit, showed a more pronounced depletion of FDG in the left midbrain at follow-up visit in comparison to baseline (*p*_FWE_ = 0.028 small-volume correction (SCV) midbrain, Fig. 2a, b). In accordance with the midbrain clusters from the comparisons to healthy controls, the latter cluster also partly covered the SN, as confirmed by a clear overlap with the SN atlas region of interest (ROI) from AAL3. Progression plots of FDG uptake in the obtained midbrain cluster for each individual patient are shown in Fig. 2b.

Similarly, voxel-wise between-group analyses of FDOPA-PET scans between patients at baseline or follow-up and healthy controls respectively showed the expected dopamine depletion in the bilateral posterior putamen (Fig. 1c). Visual comparison suggested a more severe depletion for patients at follow-up compared to healthy controls and an additional hypodopaminergic cluster in the right caudate nucleus (CAUr) was observed (Fig. 1c right). Furthermore, exported tracer uptake values from resulting clusters (follow-up<healthy controls) in the right putamen and the caudate differed between baseline and follow-up (right putamen *p* = 0.046, right caudate *p* = 0.004, Fig. 1d). The voxel-wise paired *t*-test of FDOPA-PET scans between follow-up and baseline indicated progression of dopamine depletion in the CAUr (*p*_FWE_ = 0.001 SVC striatum, Fig. 2c, d). Individual progression in FDOPA-uptake in the right caudate is shown in Fig. 2d. Both the hypometabolic midbrain and dopamine-depleted caudate clusters remained significant after using LEDD as covariate (FDG-PET, *t* = 5.01, *k* = 26, *p*_FWE_ = 0.035, FDOPA-PET, *t* = 9.40, *k* = 214, *p*_FWE_ = 0.001).

**Fig. 3:**
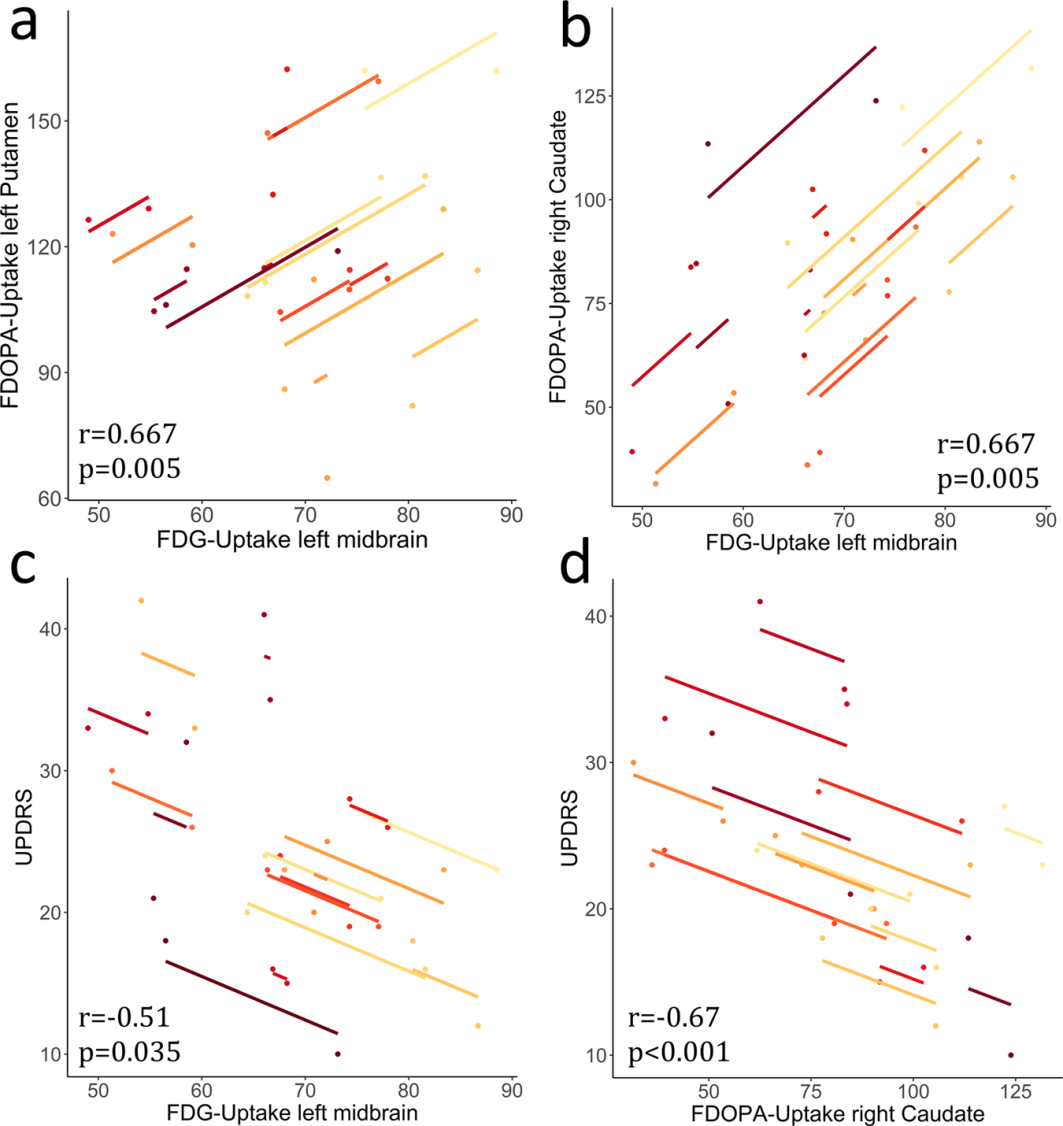
Repeated measure correlations between changes in FDG or FDOPA uptake and clinical deterioration. Rmcorr plots indicating a significantly associated decline in tracer uptake of midbrain and striatal clusters. Each colored dot represents one of the two separate variables of normalized tracer uptake (proportional scaling) for a patient; observations from the same patient are similarly colored^25^. The corresponding lines represent the rmcorr fit for each participant. Positive slopes indicate a positive linear correlation and vice versa. **a** Concomitance in progression of hypometabolism in left midbrain and dopaminergic deficit of the left putamen (*r* = 0.667, *p* = 0.005) and **b** likewise, between left midbrain and right caudate (*r* = 0.667, *p* = 0.005). Correlation between worsening in UPDRS-III OFF and changes in glucose metabolism in left midbrain (**c**) (*r* = −0.51, *p* = 0.035) and **d** increasing dopaminergic deficiency of the right caudate (*r* = −0.67, *p* < 0.001).

To verify the presumed progressive nigrostriatal pathway disintegrity analogous to our previously published findings^10^, we analyzed the interrelation over time between the decline in midbrain FDG and striatal FDOPA uptake from the clusters previously attained via follow-up vs. baseline comparisons. A repeated measure correlation (rmcorr)^25^ analysis revealed a linear correlation of FDG-uptake reduction in the left midbrain with FDOPA-uptake reduction in the left putamen (*r* = 0.60, *p* = 0.014, Fig. 3a) and CAUr (*r* = 0.67, *p* = 0.005, Fig. 3b). Decline of dopamine metabolism in the right putamen and CAUr correlated with each other (*r* = 0.60, *p* = 0.015). Aside from interregional correlations, we found associations of longitudinal changes in left midbrain hypometabolism and deterioration of motor scores such as UPDRS-III (*r* = −0.51, *p*_FDR_ = 0.047, Fig. 3c) and LBS (*r* = 0.53, *p*_FDR_ = 0.048). Caudatal dopaminergic dysfunction similarly progressed along with UPDRS-III scores (*r* = −0.67, *p*_FDR_ = 0.005, Fig. 3d) and LBS (*r* = −0.59, *p*_FDR_ = 0.037) (Supplementary Table 1).

### Progressive striatocortical dysconnectivity of dopamine-depleted areas

**Fig. 4:**
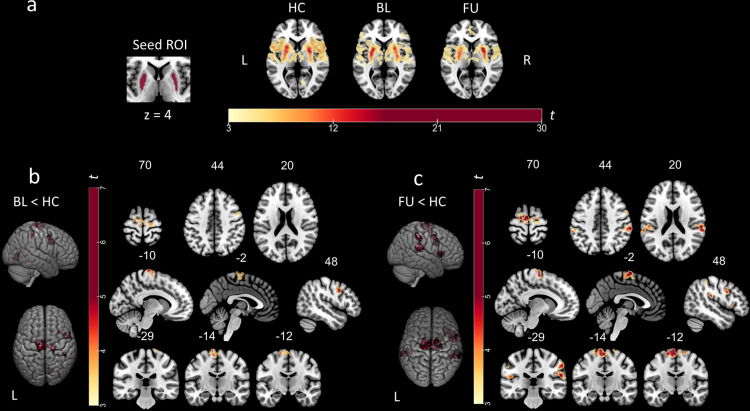
Within- and between-group striatocortical functional connectivity of dopamine-depleted putamina. *Top*: **a** Putaminal seed-to-voxel functional connectivity maps of healthy controls and patients with PD at baseline and follow-up visit. Scaled colorbar indicates *t*-values. **b** *Bottom left*: Significant differences in putaminal functional connectivity between healthy controls and patients at baseline visit. *Bottom right*: Significant differences in putaminal functional connectivity between healthy controls and patients at follow-up visit. Study-specific seed ROIs were provided by clusters derived from the FDOPA-PET comparison (FU < HC). SPM *t*-maps are overlaid on a T1 MNI template. All results were thresholded at *p*_FWE_ < 0.05 cluster level. The numbers above the slices represent MNI *x*-, *y*-, or *z*-coordinates, respectively.

First, we conducted within-group seed-based correlation analyses (one-sample *t*-test) to obtain striatocortical FC maps seeded in the specific clusters in the bilateral putamen, where dopamine depletion was most severe at follow-up in comparison to healthy controls. Striatocortical maps comprised frontoparietal regions belonging to the sensorimotor network such as the precentral gyrus, the supplementary motor area, as well as temporal and inferior parietal regions. Visual inspection suggested that connectivity patterns within the sensorimotor network were restricted in patients with PD at the baseline visit compared to healthy controls and even more so at follow-up (Fig. 4a, top).

Second, we evaluated significant differences in seed-to-voxel putaminal FC between healthy controls and both baseline or follow-up visits by applying the contrasts baseline<healthy controls and follow-up<healthy controls. The observed impairment of striatocortical coupling with regions of the sensorimotor network was more severe and widespread at follow-up than at baseline (Fig. 4, a and c, bottom). When putaminal seeds were subdivided into anterior and posterior parts, the study-specific dopamine-depleted left-anterior putamen showed more widespread changes in FC to cortical regions at follow-up visit in comparison to healthy controls, than the left posterior putamen (see Supplementary Figs. 1–2 and Supplementary Table 3). Nevertheless, both seeds revealed significant reductions in FC to regions of the sensorimotor network at baseline and follow-up visit in comparison to healthy controls (Tables 2 and 3).

**Table 2:** Results of PET neuroimaging analyses.

Modality [contrast]	Region	MNI coordinates	Statistics		Cluster size
		*x*/*y*/*z*	*t*-value	*p*-value (FWE)	
FDG-PET					
[BL < HC]	Left midbrain	−12/−20/−20	4.525	0.028	35
[FU < HC]	Bilateral midbrain	−6/−20/−18	5.55	<0.001	227
[FU < BL]	Left midbrain	−8/−10/−16	4.70	0.045	22
FDOPA PET					
[BL < HC]	Left putamen	−28/−8/−6	5.40	0.012	187
	Right putamen	28/−8/2	5.37	0.017	155
[FU < HC]	Left putamen	−28/−14/−2	7.69	0.003	362
	Right putamen	28/−10/2	8.59	0.009	228
	Right caudate	16/6/14	4.33	0.033	99
[FU < BL]	Right caudate	8/12/−2	9.73	0.001	220

Statistical details of PET analyses by contrast and modality. All *t*-values were calculated using SPM12. For FDG-PET, *p*-values after small-volume-correction for midbrain and for FDOPA PET after small-volume-correction for striatum are shown, respectively.

**Table 3:** Results of rs-FMRI neuroimaging analyses.

Contrast seed	Region	MNI coordinates	Statistics		Cluster size
		*x*/*y*/*z*	*t*-value	*p*-value (FWE)	
BL < HC	LGr, ICCr	14/−72/2	5.85	0.002	112
Seed: bilateral putamen	PreCGl, PreCGr, SFGl, SMAl, PostCGr	−10/−22/80	5.69	<0.001	416
	PreCGr, MidFGr	48/8/38	5.15	<0.001	205
	PostCGr, pSMGr, SPCr	42/−34/52	4.52	0.036	67
FU < HC Seed: bilateral putamen	PreCGl, PreCGr, SMAl, SFGr, SFGl, SMAr	−16/−10/70	6.29	<0.001	925
	IFGopr, FOr	58/18/8	5.98	0.005	93
	ICr, FOr	32/22/6	5.80	0.002	110
	aSMGr, pSMGr, PostCGr, SPCr	58/−36/50	5.73	<0.001	332
	PreCGr, MidFGr, IFGopr	40/10/26	5.58	<0.001	266
	POr, pSMGr, PTr, aSMGr	62/−36/20	5.53	<0.001	330
	POl, PTl, SMGl, pSMGl	−60/−32/16	5.43	<0.001	298
	aSMGl	−62/−40/44	4.59	0.012	79
FU < BL Seed: right caudate	sLOCl, SPCl, AGl, pSMGl	−30/−66/54	6.43	<0.001*	357
	MTGr, ITGr	70/−40/−10	5.88	0.015*	44
	FPl	−50/42/4	5.30	0.032*	33
	ITGl, MTGl	−56/−46/−16	5.22	0.001*	71
	FPl, MidFGl	−46/40/22	5.06	0.032*	33

Statistical details of rs-fMRI analyses by contrast and seed volumes. Analyses were conducted in CONN as seed-based correlations with bilateral putamina or right caudate nucleus from FDOPA PET comparison (FU < HC; FU < BL) as seeds and compared by unpaired/paired *t*-tests.

*AGI* angular gyrus left, *ICCr* intracalcarine cortex right; *ICr* right Insula, *IFGopr* inferior frontal gyrus pars opercularis right, *ITGr* inferior temporal gyrus right (temporo-occipital); *FOr* frontal operculum cortex right, *FPl* frontal pole left, *LGr* lingual gyrus right, *MidFGl* middle frontal gyrus left, *MidFGr* middle frontal gyrus right, *MTGl* mid temporal gyrus left (temporo-occipital), *MTGr* mid temporal gyrus right (temporo-occipital), *POl* parietal operculum cortex left, *POr* parietal operculum cortex right, *PostCGl* postcentral gyrus left, *PostCGr* postcentral gyrus right, *PreCGr* precentral gyrus right, *PreCGl* precentral gyrus left, *PTl* Planum temporale left, *PTr* planum temporale right, *SFGI* superior frontal gyrus left, *SFGr* superior frontal gyrus right, *sLOCl* superior lateral occipital cortex, *SMAl* supplementary motor area left, *SMAr* supplementary motor area right, *aSMGl* supramarginal gyrus left anterior, *aSMGr* supramarginal gyrus right anterior, *pSMGl* supramarginal gyrus left posterior, *pSMGr* supramarginal gyrus right posterior, *SPCl* superior parietal cortex left, *SPCr* superior parietal cortex right, *STGl* superior temporal gyrus left, *STGr* superior temporal gyrus right.

*thresholded at cluster-level *p*_FDR_ < 0.05.

Third, direct comparisons of FC maps from striatal clusters, in which significant differences in dopamine activity were identified at follow-up, were performed between both visits. For putaminal clusters, in which a significant dopaminergic deficit was identified at follow-up compared to healthy controls, a paired *t*-test between follow-up and baseline revealed no significant differences in FC, which survived correction for multiple comparison.

**Fig. 5:**
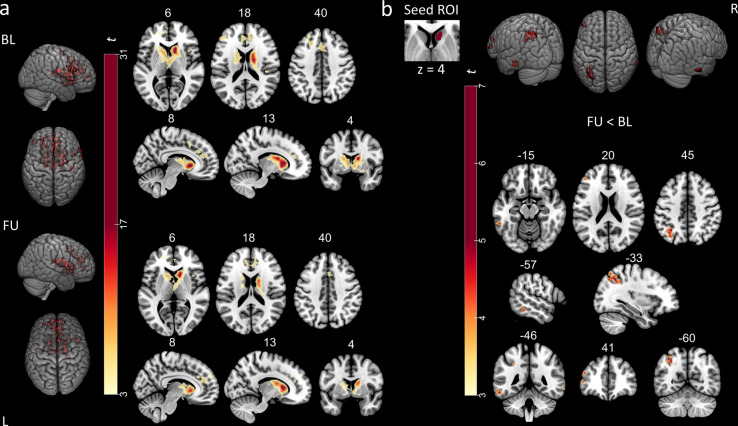
Direct comparison of caudato-cortical functional connectivity between follow-up and baseline. **a** Caudatal seed-to-voxel functional connectivity maps of patients with PD at baseline (*top*) and follow-up visit (*bottom*). Scaled colorbar indicates *t*-values. All results were thresholded at *p*_FWE_ < 0.05 cluster level. **b** Significant differences in caudatal functional connectivity between patients at baseline and follow-up visit. Study-specific seed ROI was provided by the cluster derived from the FDOPA-PET comparison (FU < BL) SPM *t*-maps are overlaid on a T1 MNI template. All results were thresholded at *p*_FWE_ < 0.05 or *p*_FDR_ < 0.05 cluster level, respectively. The numbers above the slices represent MNI *x*-, *y*-, or *z*-coordinates, respectively.

The within-group correlation analysis with caudate seed cluster, in which significant dopaminergic deficits were observed between both visits, revealed cortical clusters markedly reduced in size at follow-up than at baseline, suggesting gradual impairment of caudato-cortical connectivity (Fig. 5a). Finally, a direct comparison between baseline and follow-up was performed with a repeated measure design and the contrast follow-up<baseline. The direct comparison between both visits revealed reduced FC between the right caudate and a parietal cluster comprising the left superior lateral occipital cortex (sLOCl), left angular gyrus (AGl), left superior parietal cortex (SPCl) (*p*_FWE_ < 0.001, Fig. 5b) and the left mid temporal gyrus (MTGl, *p*_FWE_ = 0.003). When applying a more liberal threshold, additional clusters in the right inferior and mid temporal gyrus (ITGr, MTGr, *p*_FDR_ = 0.015), left frontal pole (FPl) and the left mid frontal gyrus (MidFGl, *p*_FDR_ = 0.032) were observed. Nearly identical clusters were observed when including LEDD changes as covariate. At baseline visit, the right caudate seed exhibited increased FC to a cluster located in the sLOCl in PD patients in comparison to healthy controls (*p*_FWE_ = 0.004, Supplementary Fig. 6 and Supplementary Table 3).

In a cross-modality investigation, we observed that a decline in FDOPA-metabolism in the right caudate was concomitant with a decrease of its functional coupling with SPCl (*r* = 0.82, p < 0.001), MTGl (*r* = 0.79, *p* < 0.001), ITGr (*r* = 0.82, *p* < 0.001), MidFGl (*r* = 0.72, *p* = 0.002) and to the FPl (*r* = 0.84, *p* < 0.001). To examine whether changes in interregional correlation were also accompanied by altered cortical metabolism, we conducted rmcorr analyses between caudato-cortical connectivity changes and decreases in FDG-metabolism in these clusters, which revealed an association of decline in FC and cortical tracer uptake in the ITGr (r = 0.69, p = 0.002).

### Effect of changes in functional connectivity on clinical progression

To ascertain the clinical implications of progressive striatocortical hypoconnectivity, we calculated rmcorr between Fisher-transformed ROI-to-cluster connectivity values and decline in clinical scores between both visits. This analysis yielded that decreasing functional coupling between the right caudate and all cortical clusters, in which decreased connectivity was observed at follow-up compared to baseline, was longitudinally associated with worsening in akinetic-rigid symptoms measured by the ARS subscore (SPCl, *r* = −0.66, *p*_FDR_ = 0.011, MTGl, *r* = −0.62, *p*_FDR_ = 0.011, ITGr, *r* = −0.52, *p*_FDR_ = 0.047, MidFGl, *r* = −0.70, *p*_FDR_ = 0.009, FPl, *r* = −0.53, *p*_FDR_ = 0.047). Similarly, correlations of these FC values with overall UPDRS-III OFF scores, UPDRS-III OFF unilateral body scores, and Freezing-of-Gait Questionnaire (FOG-Q) were observed in a repeated measures design (see supplementary material).

## Discussion

The results of this longitudinal multimodal imaging study were threefold: first, we identified concomitant longitudinal changes in metabolic midbrain activity and striatal dopaminergic dysfunction indicative of progressive nigrostriatal degeneration. Second, with these hypodopaminergic clusters as seed volumes for FC analyses, we revealed progressive striatocortical dysfunction above all in sensorimotor areas. Third, we rendered evidence for changes in spatial patterns during the course of PD, with an anterior-posterior gradient of dopamine dysfunction and striatocortical hypoconnectivity. Multimodal imaging progression markers were paralleled by clinical deterioration in the observed period. Thus, our findings corroborate the notion of a network-dependent degeneration in PD, encompassing distinct levels of neural dysfunction, thereby not only confirming our previous results but above all providing novel insights into its longitudinal course.

The importance of nigrostriatal dysfunction in PD is evidenced by clinicopathological correlations. While not an exclusive pathomechanism, ventrolaterally accentuated neural cell loss in the SN conform with motor disturbances^26^. Neuromelanin-sensitive MRI^13^ of the SN has been discussed as potential progression marker according to its relationship with disease duration^27,28^. Alternatively, PET imaging may ascertain nigrostriatal degeneration over the course of PD. By demonstrating progressive hypometabolism we confirmed a hypothesis derived from our previous results^10^. At follow-up, clusters encompassed large portions of the bilateral SN^29^, supporting our previous conclusion that they represent metabolic consequences of cell loss in high energy consuming populations. Hypometabolism in the midbrain or specifically SN has also been reported recently in FDG-PET studies in animal models of PD^30,31^. Strikingly, our results indicate shifts of hypometabolism towards the medial and ventral SN, which degenerate after the lateroventral parts^32^. Implications of this reduced midbrain metabolism could be underpinned clinically, as FDG uptake differences between visits strongly related to the elapsed time period and increasing motor symptom burden.

The disintegration of the SN is fundamental for the emergence of PD and yet little is known on network-level repercussions following the degeneration of its neurons. Comparative studies have shown that patients have lower striatal dopamine metabolism than healthy subjects, particularly in the posterior putamen, due to loss of nigral cells^17^. In a similar vein, an anterior-posterior gradient with a less severe and later involvement of anterior striatal portions in PD has been posited^15,33^. In our data, we found support for this notion, as the right caudate nucleus was additionally involved at follow-up in comparison to baseline and to healthy controls and as there were no differences in putaminal FDOPA uptake between both visits. Effects between visits were most pronounced in the right caudate and these changes in normalized FDOPA uptake correlated with both the increasing disease duration and worsening in UPDRS-III scores. Consistent with our findings, the caudate nucleus’ significance for disease progression is underpinned by associated atrophy^34^, increased iron content^28^ and gradual dopamine depletion^35^ as detailed in previous studies. Considering the latter studies, our results highlight the inestimable value of combining different imaging techniques for capturing direct consequences of degeneration in a “disease hub” such as the SN.

Applying different imaging techniques together offers an intriguing possibility to unravel the interdependencies between regions involved in widespread degenerative diseases^10^. In our current work, a cross-modality approach enabled us to show increasing dopaminergic deficits in the putamen and caudate relating to progressive FDG hypometabolism in the left midbrain in a repeated measures design. Although nigrostriatal connections are not exclusively unilateral^36,37^, this strong association could arguably be more associative than referring to actual anatomical pathways. Still, this might render additional evidence for a pronounced dopamine depletion in more ventral areas secondary to midbrain degeneration.

Although FC in PD has been subject to extensive investigations, reliable insights about its changes over time remain scarce. A solid body of literature describes aberrant temporal coupling within striatal structures and with widespread brain regions in PD patients^10,18,38,39^. To the best of our knowledge, the only two longitudinal studies published so far were restricted to one or two modalities and describe abnormal FC in posterior cortical areas^40^ and widespread, progressive impairment in coupling of the basal ganglia within the sensorimotor network^41^. In the current study, reduced FC of the posterior putamen to sensorimotor areas at baseline^10^ was not only replicable but also showed progression of hypoconnectivity to regions including the supplementary motor area, the precentral gyrus, and the parietotemporal area. In other words, more widespread cortical clusters with hypoconnectivity emerged at follow-up visit. In support of the idea of an anterior-posterior gradient of degeneration, we found that striatocortical connectivity of the caudate differed significantly at follow-up in comparison to baseline. Furthermore, with respect to healthy controls, the left anterior putamen exhibited more widespread striatocortical hypoconnectivity at follow-up. In agreement with these PET and rs-fMRI observations, previous rs-fMRI studies were likewise indicative of an anterior-posterior gradient and therefore of striatal dysconnectivity to different areas^38^. Furthermore, we found further evidence for a potentially compensatory role^42^ of the caudate in earlier disease stages, as the seed in which a significant dopaminergic deficit was observed at follow-up compared to baseline, exhibited an increased FC to a cluster in the iLOCl, which showed a high spatial correspondence with the corresponding dysconnectivity of the same seed observed when comparing both visits.

Among others, one simplified model suggests that basal ganglia are functionally segregated with a tripartite division into motor, associative and limbic loops. Each of these loops includes subdivisions of the striatum as evidenced by functional imaging^43^. In contrast to the putamen, the caudate shows more interaction with frontal and limbic regions^38,39,44^ as seen in our cohort for the dopamine-depleted right caudate. Nonetheless, functional connections to sensorimotor regions exist as well^39,44^, whereas their clinical relevance remains elusive. Some authors reasoned implications for gait disturbances^35^ as well as for memory and attention deficits^38,44^. In this study, FC analysis of the caudate revealed the strongest decreases to frontal and temporoparietal regions between the visits, which were associated with worsening of akinetic-rigid symptoms and therefore indicative of an involvement in increasing motor burden. Pasquini et al.^35^ described gait disturbances predicted by early caudatal dysfunction, which goes beyond the scope of this study which did not specifically address gait. However, our results also support an involvement in gait difficulties since significant changes in FOG-Q were observed between both visits and were moderately associated with dopamine depletion in the right caudate and its connectivity to the SPCl^45^.

But leaving motor symptoms aside, reduced caudatal FC to frontal areas of the default mode network seems to impair cognitive performance of patients suffering from PD^44^, and dopaminergic dysfunction has been shown to predict future cognitive decline^35^, putting our results into a more holistic perspective of PD. However, no significant progression of cognitive decline was observed in our study. One may infer relatively short intervals between visits as well as early disease stages as a possible explanation so that this hypothesis awaits further confirmation in more severely affected patients. It also remains speculative if the encountered alterations in caudate function and FC could be the herald of future cognitive decline.

One of the great strengths of this study was the appliance of different imaging techniques over the course of the disease on a well-characterized group of PD patients. Thus, in a final step FC values between clusters of dopaminergic deficiency and cortical clusters were subjected to exploratory correlation analyses. In line with previous results, a close relationship between striatal dopaminergic integrity and functional coupling was traceable^41^. Yet, unlike results from Li et al.^41^, our approach poses important advantages, insofar as that we could select study-specific dopamine-depleted structures as seeds instead of general presynaptic function. Furthermore, we demonstrated a correlation between striatocortical FC and reduced cortical FDG uptake^10^ indicating cortical metabolic consequences of reduced coupling to subcortical areas such as the right caudate. This may be seen as support for the hypotheses of a gradual spreading of disease activity with secondary degeneration. Our results may therefore offer an explanation for the plethora of studies showing cortical atrophy in specific networks^11,12,46,47^ as these areas depend upon intact connectivity to the basal ganglia^12^.

Despite its advantages in multimodality, the results of this study are subject to some limitations. First and foremost, the rather small sample size may hamper generalizability, but only a subset of patients was available for follow-up scans. Second, control subjects were only exposed once to the imaging protocol to minimize their radiation exposures. Lastly, relatively short time between measurements of approximately 14 months, reduced the possibility to assess cognitive changes, despite our findings in cortical metabolism and FC. Otherwise, noise due to the experiment itself or resulting from data acquisition needs to be born in mind in longitudinal imaging studies. We tried to counteract potential sources of bias with standardized instructions for the rs-fMRI measurements and PET acquisitions, and as they were performed by the same experimenter and all scans occurred at the same time of the day. Strong relationships between elapsed time and disease burden and neurobiological changes observed with all imaging modalities underscore the assumption that these are actual changes in disease progression and not just substantial variations within comparable disease stages. Another limitation may be the selection of non-motor symptom questionnaires, which prevented a more precise differentiation of autonomic aspects and the occurrence of, e.g., rapid eye movement (REM)-sleep behavior disorder to enable distinctions based on the current perspective of “brain-first” vs. “body-first”^8^. However, in the current study, PD patients progressed mainly in motor aspects while there was no relevant progression in overall non-motor symptoms. The exclusive finding of worsening of sleep disturbances might be mainly attributed to worsening of motor symptoms at night, which is also reflected by a high correlation with the UPRDS scores.

In summary, the present study offers a multimodal characterization of longitudinal network degeneration in a well-characterized cohort of PD patients. On the basis of a hypometabolic midbrain cluster^10^, we could demonstrate network-dependent degeneration over time, including not only a relationship between progressive midbrain hypometabolism and a concomitant change in striatal dopaminergic activity, but also associated progression of striatocortical hypoconnectivity to areas primarily involved in sensorimotor processing. Our results provide evidence for a more pronounced dopaminergic deficit and reduction of striatocortical FC of anterior striatal regions during disease progression, which supports the hypothesis of an anterior-posterior gradient of functional dysintegrity in PD. Finally, the results of this study corroborate the usefulness of multimodal imaging protocols to enhance our understanding of pathophysiological concepts of PD at a network level and may serve to develop markers for objective tracing of disease progression.

## Methods

### Standard protocol approvals, registration, and patient consents

For this follow-up examination, 17 PD patients from the initial cohort (25 healthy controls, 60 patients), agreed to participate in this multimodal longitudinal imaging study (cf. https://gepris.dfg.de/gepris/projekt/233511284) at the University of Cologne. All participants declared written informed consent in conformation with the Declaration of Helsinki. Approval was given by the ethics committee of the Medical Faculty of the University of Cologne (ethical clearance number EK12-265). Permission to apply radiation in patients and healthy controls was given by the Federal Bureau for Radiation. Patients were diagnosed according to UK-Brain-Bank criteria and were recruited via the neurological outpatient clinic at the University Hospital of Cologne and neurological practices. Healthy control subjects were recruited by word-of-mouth advertising. Exclusion criteria included: age of less than 40 years, suspected atypical parkinsonian syndromes, advanced parkinsonism (i.e., Hoehn and Yahr stage > 3)^48^, concomitant neurological disorders, safety concerns for MRI scanning and signs of dementia. The latter was excluded according to criteria published by the Movement Disorder Society by using a neuropsychological test battery and an assessment of the patient’s ability to manage daily life^49^.

The study protocol has been detailed in previous publications^10,50^. Briefly, motor symptom burden was determined using the UPDRS-III in the OFF-state^20,51,52^. Subscores for tremor, akinetic-rigid symptoms, hemibody affection, and postural instability and gait (PGS) were calculated from respective items. LEDD was calculated based on a common standard^53^. Furthermore, the FOG-Q^54^ was applied, and global cognitive functioning evaluated with a neuropsychological test battery including at least two tests for each of the following domains: attention, memory, language, executive function (Supplementary Table 2). MMSE and PANDA were applied as an additional measures of global cognitive function^55^. Beck’s Depression Inventory-II (BDI-II)^56^ served to screen for depressive symptoms. In addition to clinical examinations, the protocol included acquisitions of FDOPA- and FDG-PET and rs-fMRI (see below). Along with the 17 patients, 14 healthy control subjects received MR-imaging and both PET acquisitions. All applicable clinical assessments and questionnaires were administered to the control group alike.

### Neuroimaging data acquisition and (pre-)processing

To ensure the highest levels of comparability, all imaging acquisitions followed a strict protocol, which was defined a priori. In addition to a strict abstinence from parkinsonian medication of 12 h for levodopa and 72 h for dopamine agonists, imaging was performed at the same time of the day and on the identical machines following the same protocols (for detailed description of OFF-state specification and imaging protocol cf.^10,50^)

#### PET imaging

Both PET scans were acquired in a high-resolution research tomograph (ECAT, HRRT, Siemens, Erlangen, Germany) at the Max-Planck Institute for Metabolism Research, Cologne. Subjects were asked to discontinue their medication and were scanned in a supine position. Rigid body transformation ensured motion correction of PET frames and the last four to nine (FDOPA-PET) or three to six (FDG-PET) frames were averaged for further analyses. Averaged PET images were co-registered to subject’s fMRI data while non-linear registration ensured normalization to the respective template in Montreal Neurological Institute (MNI) space^57,58^. Normalized PET images were finally smoothed with a 6 mm full width at half maximum (FWHM) isotropic kernel and intensity normalized by using proportional scaling in SPM12 (www.fil.ion.ucl.ac.uk/spm/software/spm12).

#### Resting-state fMRI

Structural and rs-fMRI were acquired on a 3 T Siemens Magnetom Prisma scanner (software system: syngo MR D13D). For details of the structural sequence see Ruppert et al.^10^. For rs-fMRI, an echo-planar imaging sequence with the following configuration was used: TR = 776 ms, TE = 37.4 ms, 617 timepoints, 72 slices at a voxel size of 2 × 2 × 2 mm. Preprocessing was carried out using the default pipeline of the SPM toolbox CONN v17^59^, including motion correction, direct segmentation and normalization into MNI space, smoothing with a 5 mm (FWHM) isotropic Gaussian kernel, temporal band-pass filtering (0.01−0.1), linear detrending, and anatomical component-based noise correction^60^.

### Statistical analysis

#### Neuroimaging

All analyses were conducted in SPM12 or the CONN toolbox^59^. PET-tracer uptake between baseline respectively follow-up timepoints and healthy controls was compared via voxel-wise two-sample *t*-tests on a whole-brain level with proportional scaling. For direct comparisons between baseline and follow-up timepoints, voxel-wise paired *t*-tests were conducted. In both cases, additional analyses with SVC for the bilateral midbrain obtained from TD-ICBM atlas^61^ or the bilateral striatum obtained from AAL3^29^, were applied. Significance levels for PET-analyses were set at *p* < 0.05 with cluster-level FWE correction for multiple comparisons. In both cases, an additional analysis with LEDD as covariate was conducted. Mean tracer uptake of ROIs were then exported via MarsBaR (http://marsbar.sourceforge.net)^62^ and utilized for further statistical analyses and grouped boxplots. Binary masks of clusters representing between-group differences in the PET modalities served as seeds for FC analysis. For a meticulous subdivision of striatal structures and to account for a presumed anterior-posterior gradient, we used the parcellation of the Melbourne Subcortical Atlas^63^ to further subdivide clusters obtained from FDOPA-PET imaging, which resulted in study-specific ROIs of posterior (pPUTl, pPUTr) and anterior putamen (aPUTl, aPUTr), as well as posterior (pCAUr) and anterior caudate nucleus (aCAUr).

For rs-fMRI, seed-based correlation analyses on a whole-brain level with the previously obtained ROIs as seed volumes were conducted. Individual seed-to-voxel FC maps were generated using mean timeseries from respective seeds and a bivariate correlation approach in a first level analysis. Second level analyses consisted of three steps: (i) group FC maps were generated with a one-sample-*t*-test, (ii) between-group FC differences resulted from *t*-tests between either of the two timepoints and healthy controls, and (iii) between-group changes over time (baseline vs. follow-up) resulted from a repeated-measure design. Results were considered significant at *p*_FWE_ < 0.05 on cluster level. For paired *t*-tests a more liberal threshold was applied (FDR). Axial, sagittal, and coronal slices and 3D views of obtained statistical maps were created using MRIcroGL.

#### Clinical and behavioral data

Statistical analyses of demographic, clinical, and behavioral variables were conducted in R^64^ with group comparisons using Welch’s *t*-tests, Mann–Whitney-*U*-tests or Chi-Squared-tests, as appropriate. For correlations, Pearson’s *r* or Spearman’s *ρ* were used. To account for the specific burden of tremor, akinesia and rigidity, laterality and gait disturbances in a given patient, we calculated respective subscores of the UPDRS-III items. Hemibody scores (RBS, LBS) were calculated from items 20−26 (0 to a maximum of 36 points) for left and right side respectively. ARS encompassed the items 18,19,22,27−31 leading to a maximum of 48 points. TS was assessed as the sum of items 20 and 21. Finally, a postural-instability-gait score (PGS) was computed from items 27−30. Linear associations between longitudinal changes in respective clinical measures with changes over time and imaging parameters were investigated by means of repeated measure correlations (*rmcorr* package)^25^ with a false-discovery-rate correction for multiple comparisons. To account for interdependency, this approach relies upon a repeated-measure analysis of covariance and is expressed by Eqs. (1) and (2) (*j* = participant, *i* = trial):

1 $${\mathrm{Measure}}1_{ij}^\prime = \overline {{\mathrm{Measure}}1} _j + {\mathrm{Subject}}_j + \beta \left( {{\mathrm{Measure}}2_{ij} - \overline {{\mathrm{Measure}}2_j} } \right)$$

with *j* being a participant and *i* denoting a trial. Thus, measure1_ij_’ represents the predicted *y*-value of Measure1 for subject *j* in trial *i* whereas $\overline {{\mathrm{Measure}}1} _j$ is the mean of Measure1 for all trials of subject *j* and *β* represents the overall common slope. Consecutively, the rmcorr coefficient *r*_rm_ can be estimated using the sums of squares values (SS) according to^25^:

2 $$r_{{\mathrm{rm}}} = \sqrt {\frac{{{\mathrm{SS}}_{{\mathrm{Measure}}}}}{{{\mathrm{SS}}_{{\mathrm{Measure}}} + {\mathrm{SS}}_{{\mathrm{Error}}}}}}$$

#### Cross-modality correlation analysis

Cross-modality correlations were performed at several levels. First, on nigrostriatal level between FDG uptake in left midbrain and FDOPA uptake in striatal clusters. Second, on a striatocortical level between striatal FDOPA uptake and striatocortical FC values and finally, on a cortical level, linking striatocortical FC with cortical FDG uptake of affected regions. Correlations between normalized PET uptake values, extracted via MarsBaR from individual clusters resulting from PET comparisons and respective ROI-to-Cluster FC values were calculated in R. The latter were directly exported from significant clusters for each subject using the CONN toolbox. Interrelations between longitudinal changes in tracer uptake and FC were analyzed using rmcorr^25^.

## Supplementary information

Supplements

## Data Availability

The data set analyzed in the present study will be made available by the corresponding author upon reasonable request.

## Code availability

The code for statistical analysis in R will be made available by the corresponding author upon reasonable request.
