# Supplementary material for: Longitudinal trimodal imaging of midbrain-associated network degeneration in Parkinson’s disease
Source: NPJ Parkinsons Dis. 2022 Jun 22;8:79. doi: 10.1038/s41531-022-00341-8 (PMC9218128; doi:10.1038/s41531-022-00341-8)
Supplement: Supplementary file 1 — Supplements [file 41531_2022_341_MOESM1_ESM.pdf]

Supplementary Table 1: Correlations between imaging findings and clinical parameters

Clinical Parameters	Modality	ROI's	r-value	p-value (FDR-corrected)
UPDRS-III OFF	FDG-PET	Left Midbrain	-0.54	0.074
	FDOPA-PET	Right Caudate	-.0.67	0.016
	rs-fMRI	SPCL	-0.76	0.003
		MTGL	-0.77	0.003
		ITGr	-0.61	0.019
		MidFGl	-0.82	0.001
		FPl	-0.66	0.011
TS	rs-fMRI	MidFGl	-0.52	0.047
FOG	FDOPA-PET	Right Caudate	-0.65	0.019
	rs-fMRI	SPCl	-0.52	0.047
RBS	rs-fMRI	MTGl	-0.63	0.016
LBS	FDG-PET	Left Midbrain	-0.53	0.048
	FDOPA-PET	Right Caudate	-0.59	0.037
	rs-fMRI	SPCl	-0.53	0.047
		ITGr	-0.61	0.019
ARS	rs-fMRI	SPCl	-0.66	0.011
		MTGl	-0.66	0.011
		ITGr	-0.52	0.047
		MidFGl	-0.70	0.009
		IFgl	-0.53	0.047
PDSS	FDG-PET	Left Midbrain	-0.59	0.035
	FDOPA-PET	Right Caudate	-0.80	0.003
	rs-fMRI	SPCL	-0.82	0.001
		MTGL	-0.69	0.010
		ITGr	-0.67	0.011
		MidFGl	-0.66	0.011
		IFGL	-0.74	0.003

Significant correlations between imaging findings and clinical parameters, that were conducted as rmcorr in a repeated measure ANOVA design. All clinical parameters with significant changes between baseline and follow-up (UPDRS-III, RBS, LBS, ARS, TS, FOG, PDSS) and all significant PET and rs-fMRI changes were included (overall 49 tests). In case of rs-FMRI, the dopamine depleted right caudate served as a seed. Correction for multiple testing was ensured using false-discovery-rate (FDR) correction with a threshold of $p_{FDR}<0.05$. Only significant results are shown in the table.

Supplementary Table 2: Neuropsychological testing

Clinical Parameters	Baseline (Mean ± SD)	Follow-up (Mean ± SD)	p-value	Test statistics
PANDA	24.1 ± 3.6	26.1 ± 2.8	**0.025**	t = -2.74
WCS	11.9 ± 3.0	10.4 ± 4.7	0.251	t = 1.20
WMS[a]	43.4 ± 10.7	42.9 ± 11.4	0.826	t = 0.22
WMS[b]	46.3 ± 6.2	51.3 ± 11.0	0.103	t = -1.73
WMS[c]	47.0 ± 12.2	47.0 ± 11.4	1.000	t = 0.00
WMS[d]	46.5 ± 7.6	47.1 ± 8.36	0.861	W = 42.50
RWT_B	54.1 ± 8.2	54.4 ± 8.1	0.950	W = 51.00
RWT_S	54.2 ± 6.5	54.5 ± 7.0	0.202	t = -1.33
RWT_setshift[a]	49.4 ± 7.7	53.2 ± 12.2	0.126	t = -1.62
RWT_setshift[b]	53.7 ± 9.6	55.2 ± 7.3	0.350	t = -1.00
RWT_profession	55.6 ± 9.8	55.9 ± 9.5	0.753	W = 47.00
RWT_animals	65.1 ± 7.4	53.1 ± 10.5	0.345	W = 59.50

Details of neuropsychological testing. Differences in variables between baseline and follow-up were analyzed with paired t-tests or Wilcoxon signed-rank tests. Tests: PANDA = Parkinson's Disease Dementia Assessment, WCS = Wisconsin Card Sorting Test, WMS = Wechsler Memory Scale ([a]= spatial forward, [b]= spatial backward, [c]= digit forward, [d]= digit backward), RWT = Regensburger Verbal Fluency Task (Letters B, S, professions, and animals, setshift[a]= phoniatric setshifting, setshift[b]= semantic setshifting)

Supplementary Table 3: Results of rs-FMRI Neuroimaging Analyses

Contrast<br>Seed	Region	MNI Coordinates<br>x/y/z	Statistics<br>T-value	 p-value (FWE)	Cluster size
BL<HC<br>aPUTl	PreCGl PreCGr PostCGl	20/-24/68	5.24	0.022	68
	PreCGr	-4/-18/62	5.12	<0.001	124
FU<HC<br>aPUTl	PreCGl, SMAl, AC, PreCGr, SMAr, PaCiGr	-4/-6/42	7.08	<0.001	724
	PreCGr, SFGr	20/-10/66	5.36	<0.001	125
	PostCGr, SPLr	34/-34/56	5.27	<0.001	156
	PreCGl, PC	-10/-28/50	5.25	0.024	64
	POl, PTl, HGl	-46/-30/16	5.13	<0.001	126
	aSMGr, pSMGr	64/-30/42	4.98	0.007	79
	Right Putamen	26/0/12	4.91	0.047	56
	ICr, FOr	32/22/10	4.83	0.034	60
	POr, pSMGr, PTr	64/-34/18	4.35	0.024	64
BL<HC<br>aPUTr	IFGOr, IFGTriGr	58/18/8	6.91	0.010	78
	OPr, iLOCr	28/-94/2	5.75	<0.001	183
	PreCGr, MidFGr	50/8/36	5.74	<0.001	140
	PreCGl, MidFGl	-52/4/46	5.51	<0.001	161
	OPr, sLOCr	14/-88/24	5.47	0.002	98
	ICCr, SCCr, LGr	10/-70/10	5.39	<0.001	150
	iLOCr	42/-70/-6	4.57	0.002	103
FU<HC<br>aPUTr	PreCGr	54/-2/48	5.59	<0.001	236
	PreCGr	26/-24/50	5.50	<0.001	129
	aSMGr, pSMGr	52/-32/42	5.39	0.001	111
	PreCGr	42/-10/60	5.25	<0.001	137
	SMAl, SFGl, PreCGl, PreCGr	6/-20/58	5.19	<0.001	296
	COl, POl, PTl	-54/-18/18	5.19	<0.001	120
	aSMGr, pSTGr	68/-32/16	5.12	0.017	69
BL<HC<br>pPUTl	PreCGl, SFGl, PreCGr, SMAl	-12/-22/80	5.29	<0.001	304
	aSMGl, POl	-66/-34/24	5.14	0.015	77
	PreCGr, MidFGr	50/6/40	5.11	<0.001	244

	LGr, ICCr	12/-72/-8	5.03	0.004	95
	aSMGr, POr	60/-26/26	4.61	0.025	70
FU<HC pPUTl	aSMGr, PreCGl, SFGl, SFGr, PreCGr, POl, IFGOr, POr, pSMGr, SMAl, ICr, PTl, FOr, aSMGl, SMAr, PTr	-16/-8/68	6.38	<0.001	600
	ICr, FOr	30/24/6	5.66	0.005	91
	POr, aSMGr, pSMGr, PTr	64/-36/20	5.40	<0.001	197
	POl, PTl, aSMGl, pSMGl	-64/-38/22	5.09	<0.001	165
	IFGOr, FOr	50/16/2	4.68	0.020	71
	PreCGr, IFGOr	44/8/32	4.60	0.020	71
	aSMGr, pSMGr	64/-34/46	4.59	<0.001	151
BL<HC pPUTr	PostCGl	-58/-18/42	5.24	0.014	76
	LGr, ICCr	4/-76/-4	5.16	0.036	63
	PreCGl, SMAl, SFGl	8/-24/78	5.13	<0.001	200
	PreCGr, SFGr	8/-24/58	4.79	0.001	110
FU<HC pPUTr	POr, PreCGl, POl, PreCGr, PostCGl, aSMGr, SMAl, PTl, pSMGr, aSMGl, COl, PTr	-16/-18/78	6.38	<0.001	182
	POr, PTr, pSMGr, COr	48/-26/22	4.80	<0.001	173
	POl, PTl, COl	-58/-32/16	4.70	<0.001	162
	PostCGl	-60/-20/40	4.37	0.006	85
	aSMGr, pSMGr	64/-34/46	4.23	0.021	68
	PreCGr, MidFGr	52/4/48	4.18	0.002	97
BL>HC CAUr	sLOCl	-26/-72/46	5.63	0.004	77

*Statistical details of rs-fMRI analyses by contrast and seed volumes. Analyses were conducted in CONN as seed-based correlations with unilateral putamina clusters from ^{18}F-DOPA PET comparisons (FU<HC), divided into posterior and anterior parts by using the Melbourne subcortical atlas, as seeds and compared by unpaired t-tests. Additionally, significant results for the contrast BL>HC and the caudate cluster from paired ^{18}F-DOPA PET comparisons are shown. *thresholded at cluster level $p_{FWE}<0.05$.*

Abbreviations: aSMGl = anterior supramarginal gyrus left; aSMGr = anterior supramarginal gyrus right; AC = anterior cingulate cortex; CalGr = calcarine gyrus right; CAUr = right caudate; COl = central operculum left; FOr = frontal operculum right; HGl = Heschl gyrus left; ICr = insular cortex right; IFGOr = inferior frontal gyrus pars opercularis right; IFGTriGl = inferior frontal gyrus pars triangularis left; IFTriGr = inferior frontal gyrus pars triangularis right; iLOCr = inferior lateral occipital cortex right; IPCl = inferior parietal cortex left; IPCr = inferior parietal cortex right; ITGl = inferior temporal gyrus left; ITGr = inferior temporal gyrus right; LGr = lingual gyrus

right; MidFGl = mid frontal gyrus left; MidFGr = mid frontal gyrus right; MTGl = mid temporal gyrus left; MTGr = mid temporal gyrus right; OPr = occipital pole right, PaCiGr = paracingulate gyrus right; PC = posterior cingulate cortex; POl = parietal operculum left; POr = parietal operculum right, PTl = Planum temporale left; PTr = planum temporale right, PostCGl = postcentral gyrus left; PostCGr = postcentral gyrus right; PreCGr = precentral gyrus right; PreCGl = precentral gyrus left; pSMGl = posterior supramarginal gyrus left; pSMGr = posterior supramarginal gyrus right; pSTGr = posterior superior temporal gyrus right; SCCr = superior calcarine cortex right, sLOCl = superior lateral occipital cortex left; SPCl = superior parietal cortex left; STGl = superior remporal gyrus left; STGr = superior temporal gyrus right; SMAl = supplementary motor area left; SMAr = supplementary motor area right; SFGl = superior frontal gyrus left; SFGr = superior frontal gyrus right; SPCr = superior parietal cortex right; ROl = Rolandic operculum left; SPLr = superior parietal lobe right; ROI = Rolandic operculum left;

Supplementary Figure Legends:

Supplementary Figures 1-4 Within- and between-group differences in striatocortical functional connectivity of dopamine depleted anterior and posterior putamina. *Top*: **(a)** Putaminal seed-to-voxel functional connectivity maps of healthy controls and patients with Parkinson's disease at baseline and follow-up visit. Scaled colorbar indicates T-values. **(b)** *Bottom left*: Significant differences in putaminal functional connectivity between healthy controls and patients at baseline visit. *Bottom right*: Significant differences in putaminal functional connectivity between healthy controls and patients at follow-up visit. Study-specific seed ROIs were provided by clusters derived from the F-DOPA-PET comparison (FU< HC) and divided into anterior and posterior parts using the Melbourne subcortical atlas. SPM *t*-maps are overlaid on a T1 MNI template. All results were thresholded at $p_{FWE}<0.05$ cluster level. The numbers above the slices represent MNI x-, y-, or z-coordinates, respectively.

Supplementary Figure 5 Correlation between PET-tracer uptakes and functional connectivity. (a-e) Repeated measure correlations between FDOPA-uptake in right caudate and striatocortical functional connectivity. **(f)** Repeated measure correlation between striatocortical functional connectivity and cortical FDG metabolism.

Supplementary Figure 6 Increased striatocortical functional connectivity of the dopamine depleted caudate (FDOPA cluster FU<BL) in PD patients at baseline in comparison to controls. SPM t-maps are overlaid on a T1 MNI template. All results were thresholded at pFWE<0.05 cluster level. The numbers above the slices represent MNI x-, y-, or z-coordinates, respectively. The resulting cluster shows spatial overlap with the cluster in which a significantly reduced functional connectivity was observed at follow-up in comparison to baseline (Main Manuscript Figure 5B).

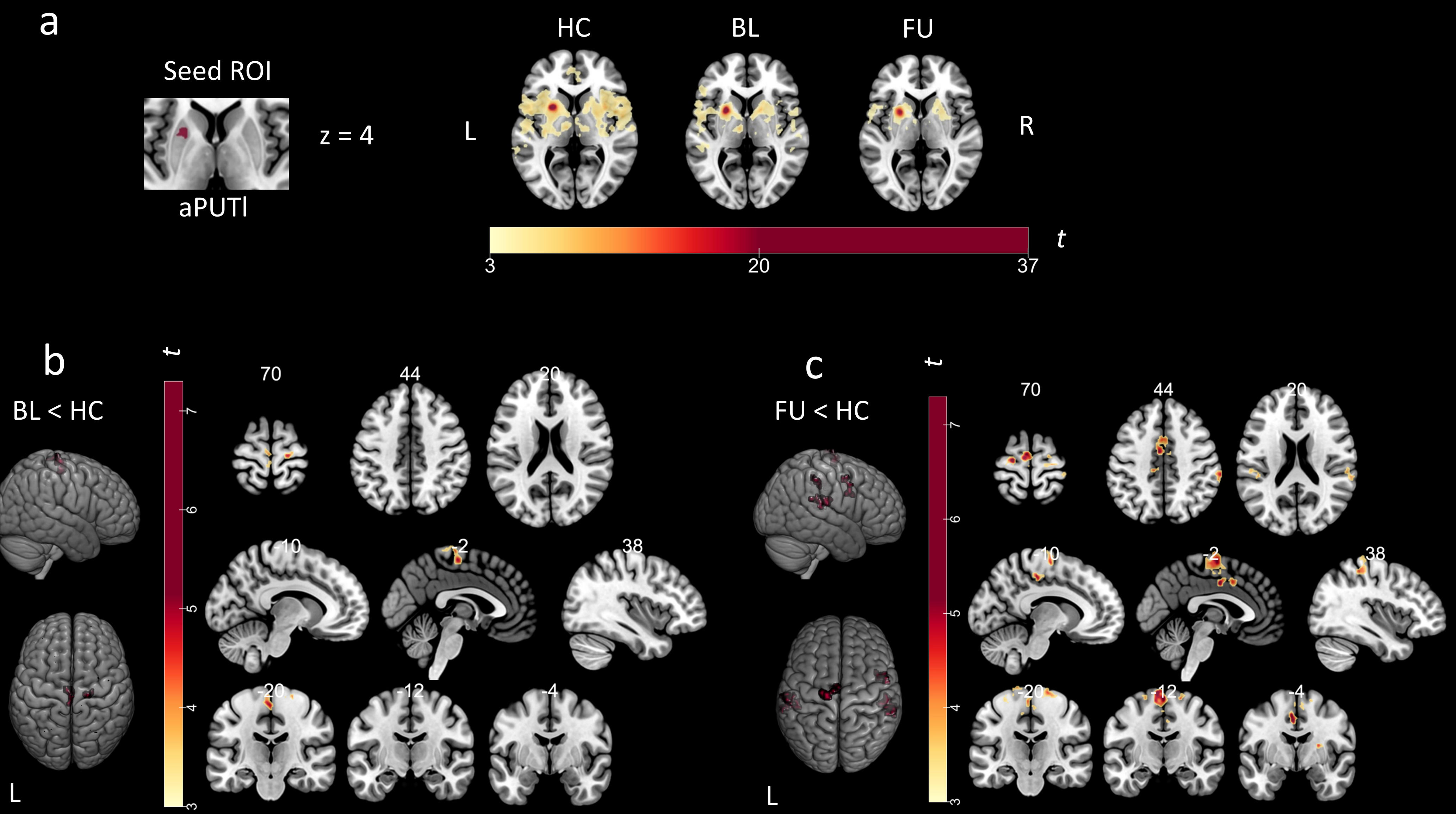
a
Seed ROI
aPUTl
z = 4
HC
BL
FU
L
R
t
3
20
37
b
BL < HC
t
70
44
20
-10
-2
38
-20
-12
-4
L
c
FU < HC
L

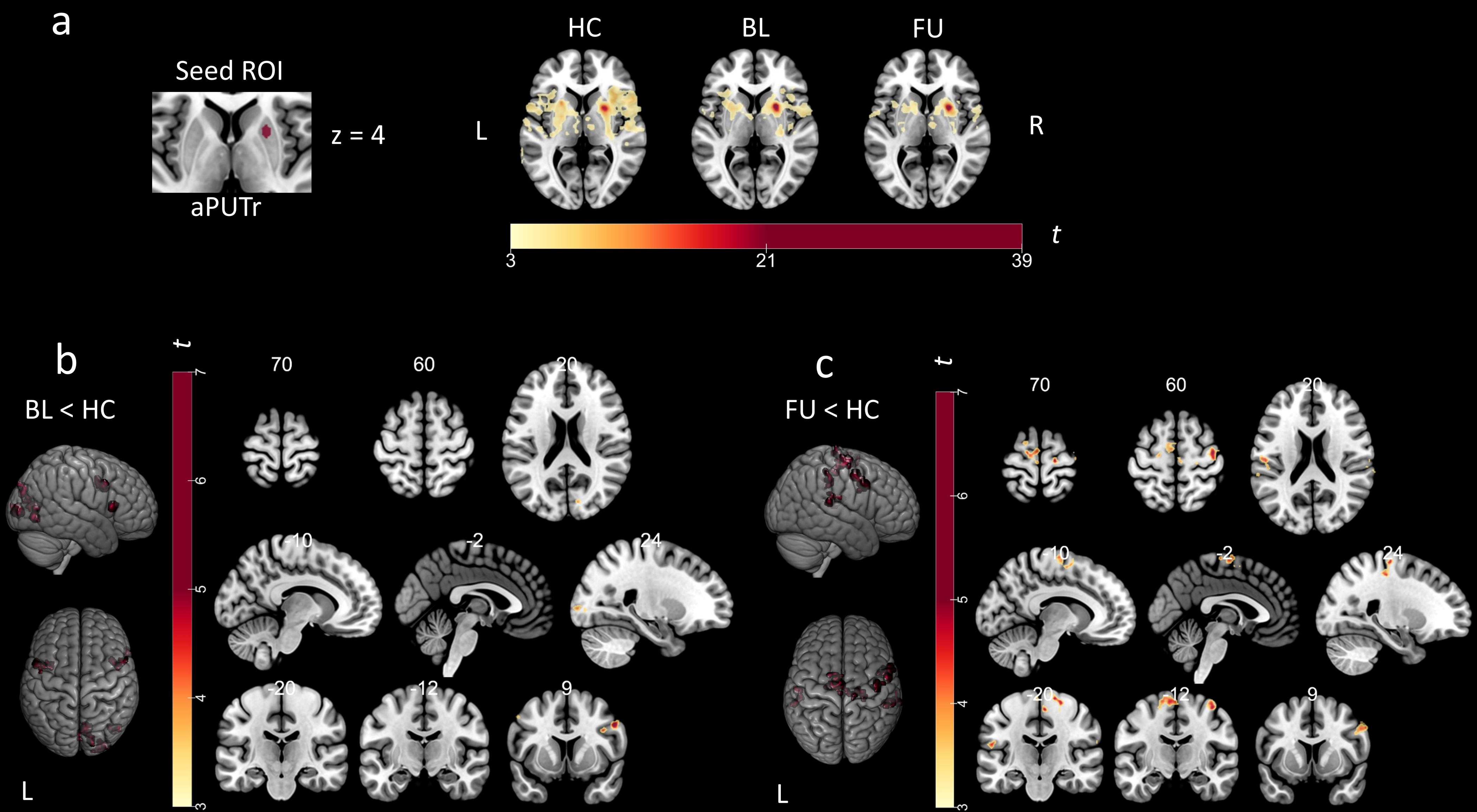
a
Seed ROI
z = 4
aPUTr
HC
BL
FU
L
R
t
3
21
39
b
BL < HC
t
70
60
20
-10
-2
24
-20
-12
9
L
c
FU < HC
t
70
60
20
-10
-2
24
-20
-12
9
L

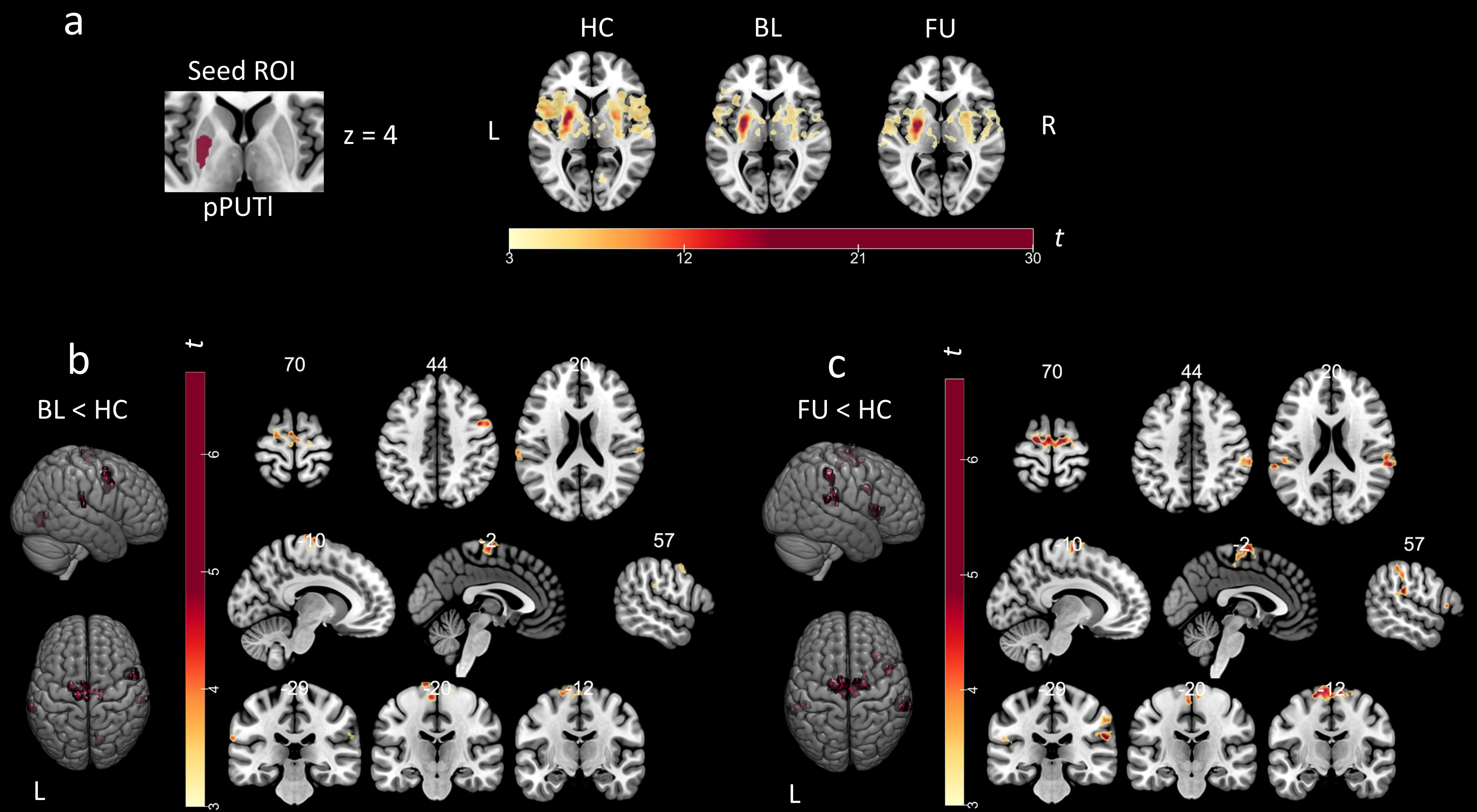
a
Seed ROI
z = 4
pPUTl
HC
BL
FU
L
R
t
3
12
21
30
b
BL < HC
t
6
5
4
3
70
44
20
-10
-2
57
-29
-20
-12
L
c
FU < HC
t
6
5
4
3
70
44
20
-10
-2
57
-29
-20
-12
L

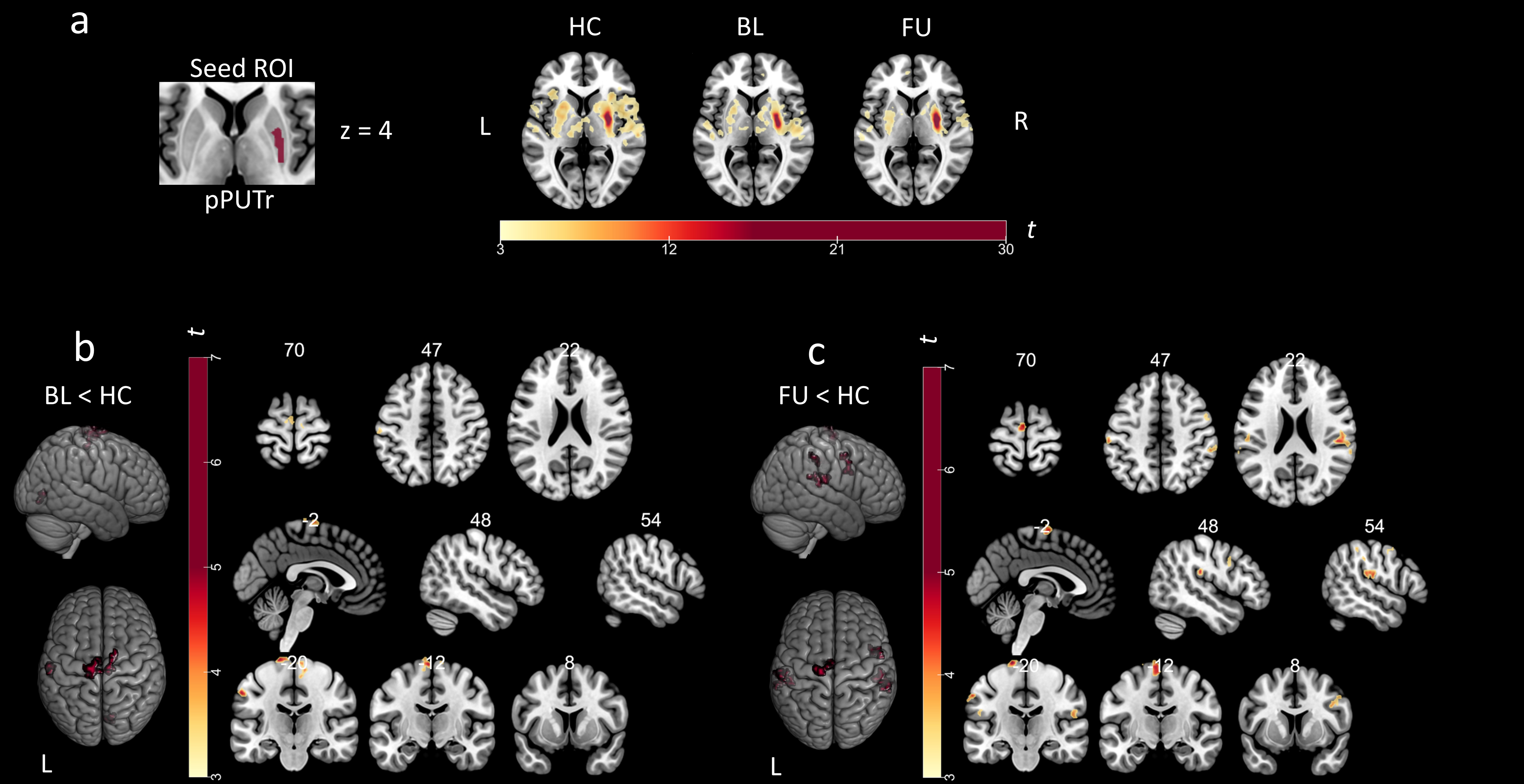
a
Seed ROI
z = 4
pPUTr
HC
BL
FU
L
R
t
3
12
21
30
b
BL < HC
L
t
70
47
22
-2
48
54
-20
-12
8
c
FU < HC
L
t
70
47
22
-2
48
54
-20
-12
8

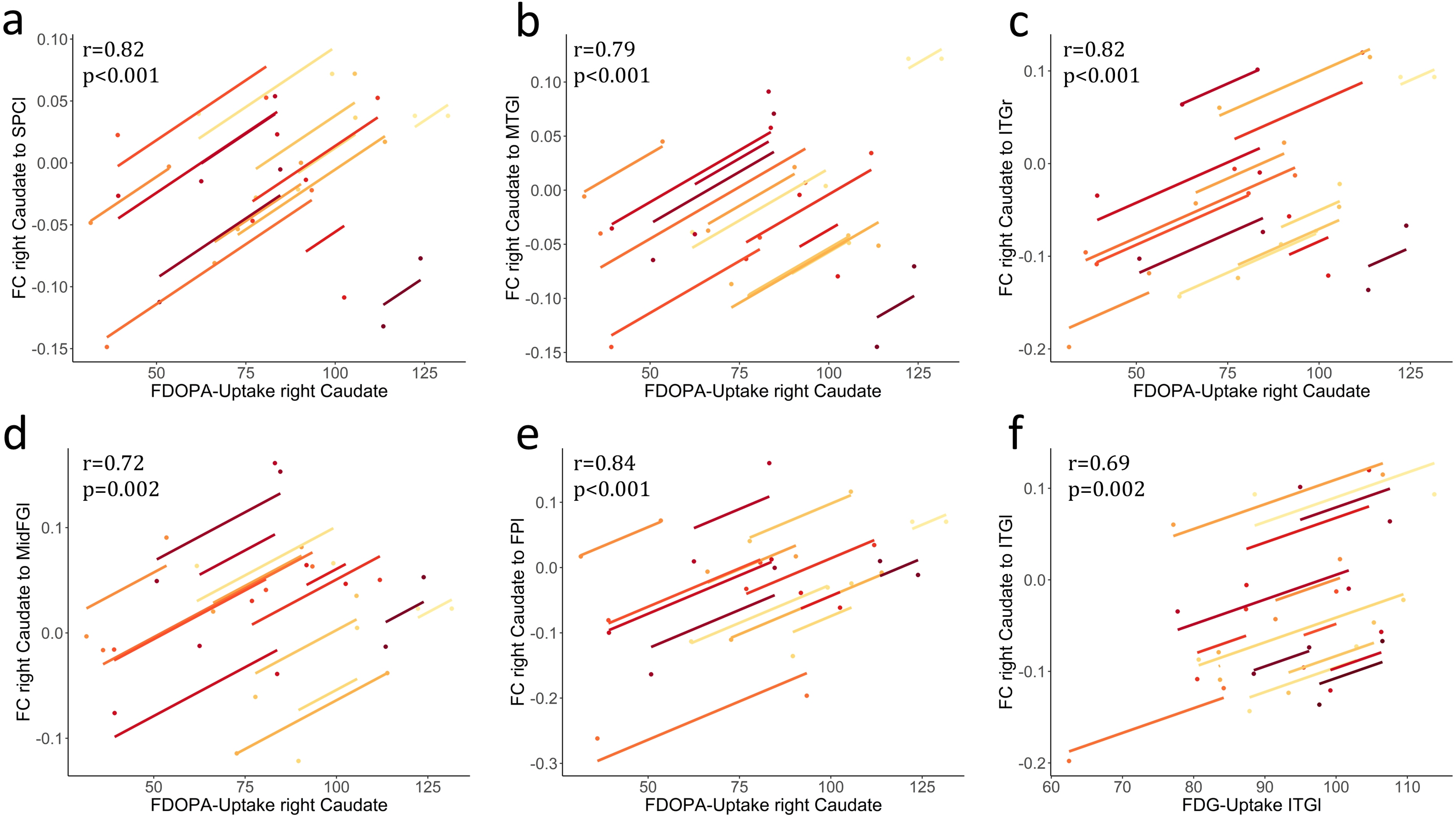
a
r=0.82
p<0.001
FC right Caudate to SPCl
FDOPA-Uptake right Caudate
b
r=0.79
p<0.001
FC right Caudate to MTGl
FDOPA-Uptake right Caudate
c
r=0.82
p<0.001
FC right Caudate to ITGr
FDOPA-Uptake right Caudate
d
r=0.72
p=0.002
FC right Caudate to MidFGl
FDOPA-Uptake right Caudate
e
r=0.84
p<0.001
FC right Caudate to FPl
FDOPA-Uptake right Caudate
f
r=0.69
p=0.002
FC right Caudate to ITGl
FDG-Uptake ITGl

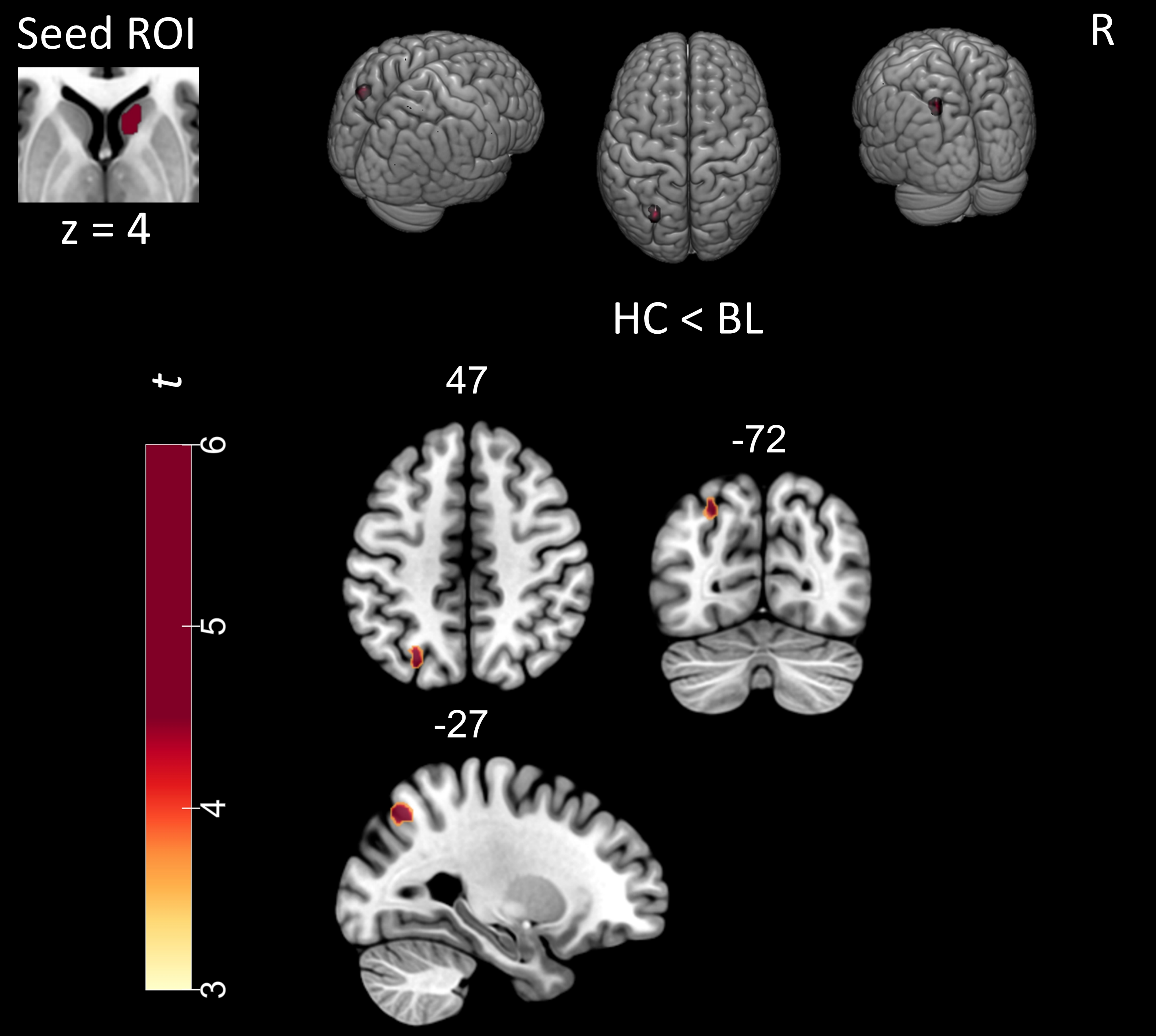
Seed ROI
z = 4
R
HC < BL
47
-72
-27
t
6
5
4
3
